# The Influence of Carbonaceous Matrices and Electrocatalytic MnO_2_ Nanopowders on Lithium-Air Battery Performances

**DOI:** 10.3390/nano6010010

**Published:** 2016-01-06

**Authors:** Alessandro Minguzzi, Gianluca Longoni, Giuseppe Cappelletti, Eleonora Pargoletti, Chiara Di Bari, Cristina Locatelli, Marcello Marelli, Sandra Rondinini, Alberto Vertova

**Affiliations:** 1 Dipartimento di Chimica, Università degli Studi di Milano, via Golgi 19, 20133 Milano, Italy; alessandro.minguzzi@unimi.it (A.M.); eleonora.pargoletti@gmail.com (E.P.); cristina.locatelli@unimi.it (C.L.); sandra.rondinini@unimi.it (S.R.); alberto.vertova@unimi.it (A.V.); 2 ISTM-CNR, Istituto di Scienze e Tecnologie Molecolari, c/o Dipartimento di Chimica, Università degli Studi di Milano, via Golgi 19, 20133 Milano, Italy; 3 Dipartimento di Scienza dei Materiali, Università degli Studi di Milano Bicocca, via Roberto Cozzi 55, 20125 Milano, Italy; gianluca.longoni@mater.unimib.it; 4 Consorzio Interuniversitario Nazionale per la Scienza e Tecnologia dei Materiali—INSTM, via G. Giusti 9, 50121 Firenze, Italy; 5 Istituto de Catalisis y Petroleoquimica, Consejo Superior de Investigaciones Cientificas, C/Marie Curie 2, L10, 28049 Madrid, Spain; cdi.bari@csic.es; 6 CNR-ISTM/ISTeM, via Fantoli 15/16, 20138 Milano, Italy; m.marelli@istm.cnr.it

**Keywords:** manganese dioxide nanoparticles, silver doping, mesoporous carbon, gas diffusion electrode (GDE), Li-air battery

## Abstract

Here, we report new gas diffusion electrodes (GDEs) prepared by mixing two different pore size carbonaceous matrices and pure and silver-doped manganese dioxide nanopowders, used as electrode supports and electrocatalytic materials, respectively. MnO_2_ nanoparticles are finely characterized in terms of structural (X-ray powder diffraction (XRPD), energy dispersive X-ray (EDX)), morphological (SEM, high-angle annular dark field (HAADF)-scanning transmission electron microscopy (STEM)/TEM), surface (Brunauer Emmet Teller (BET)-Barrett Joyner Halenda (BJH) method) and electrochemical properties. Two mesoporous carbons, showing diverse surface areas and pore volume distributions, have been employed. The GDE performances are evaluated by chronopotentiometric measurements to highlight the effects induced by the adopted materials. The best combination, hollow core mesoporous shell carbon (HCMSC) with 1.0% Ag-doped hydrothermal MnO_2_ (M_hydro_1.0%Ag) allows reaching very high specific capacity close to  1400 mAh·g^−1^. Considerably high charge retention through cycles is also observed, due to the presence of silver as a dopant for the electrocatalytic MnO_2_ nanoparticles.

## 1. Introduction

Electrochemical power suppliers, capable of storing high quantities of energy, have always been one of the most challenging topics in electrochemistry. The improvement of their efficiency, especially in terms of their energy density, could lead to the exploitation of these devices in the automotive industry and in the correct development of renewable energy sources, giving the possibility to overcome the discontinuous energy production. Recently, thanks to the exploratory work by Abraham *et al.* [1], a new device (Li-O_2_ battery) able to give high theoretical energy densities has begun to be investigated. It is a secondary battery, akin to the familiar metal-air devices, like zinc-air batteries: ((−) Li/non-aqueous electrolyte/O_2_ (air) (+)). It is noteworthy to remember that the oxidation of 1 kg of lithium releases around 12 kWh, a value comparable to the theoretical energy density of gasoline [2].

One of the most challenging features in the cathodic compartment of metal-air batteries is the composite gas diffusion electrode (GDE), which has to be permeable by oxygen in alternating directions (discharge/charge reactions). Thus, air-cathode structures and the employed material morphologies are crucial to assess from both electrochemical [3,4,5] and morphological/structural [6] techniques. Moreover, in these systems, current density, electrolyte composition and discharge products (Li_2_O_2_ (*E* = 3.10 V *vs.* Li^+^/Li) and the subsequent reduced species Li_2_O (*E* = 2.72 V *vs.* Li^+^/Li) [7,8]) are important parameters that can seriously affect the performance of the entire battery [9,10,11]. In particular, the air-cathode (the oxygen reduction site) requires a complex combination of electrocatalytic materials, supports and a current collector in order to optimize the formation of an extended triple contact and the fast and reversible formation/removal of the cathode reaction products. Recent literature studies have demonstrated how the specific capacity has a stronger reliance on the pore size distribution of the cathode material (typically graphitic materials) than a direct correlation with the total surface areas [12,13,14]. Meso- and macro-porosity allows insoluble LiO_2_ and Li_2_O_2_ discharge products to homogeneously fill the pores, whereas micro-pores become quickly top-clogged by solid particles, determining the fast decay in the active surface area of the material [15,16]. Aiming at obtaining an optimal response between discharge and charge curves, suitable electrocatalysts have to be included into the cathode structure, in order to reduce and equalize the overpotentials for the oxygen reduction/oxidation. Thus, many transition metals and their relative oxides, such as Au, Pt, NiO, Fe_2_O_3_ and Fe_3_O_4_ in aprotic media [17] and IrO_2_-SnO_2_ mixtures in alkaline protic solvent [18,19], have been investigated. Moreover, CoFe_2_O_4_ and CuO nanoparticles have been also tested giving the best capacity retention properties [17]. In this context, new approaches have been developed exploiting a solution-phase catalyst in order to catalyze the Li_2_O_2_ decomposition during the charge cycle [20,21,22]. The most promising material, in terms of performances in both oxygen reduction (discharge) and evolution (charge) and costs, seems to be manganese dioxide nanoparticles. According to the literature, MnO_2_ would ensure capacities comparable to those of platinum, letting higher capacity retention to be reached [23,24], even in the presence of non-aqueous electrolytes, widely used to prevent Li decomposition. However, these non-aqueous electrolytes can be affected by electrode surface potentials, causing a rapid degradation of the electrolyte itself and leading to other discharge products (lithium alkyl carbonates or simply Li_2_CO_3_) [25,26,27]. The usage of propylene carbonate (PC), and in general of organic carbonates, is still an open debate and studies on the mechanism and by-product formation of carbonate solvent degradation in Li/air batteries are going on [27]. To overcome this problem, aprotic electrolytes (such as DMSO or tetraethylene glycol dimethyl ether (TEGDME)) have been used lately in Li-O_2_ batteries [28,29,30]. However, also these newly-adopted solvents show some drawbacks, *i.e.*, unwanted reactions leading to the consequent formation of by-products, such as, for example, Li_2_CO_3_ and LiOH [31,32].

In the present research work, the electrocatalytic activity of different hydrothermally-synthesized MnO_2_, supported on *ad hoc* home-made mesoporous carbons, is evaluated using two different lithium-air cell configurations. Correlations between the physico-chemical characteristics of the materials, employed to prepare GDEs and the final electrical performances of the cell, are drawn. Moreover, taking into account all of the shortcomings related to the use of non-aqueous electrolytes [17,33,34], LiClO_4_ in PC (a low cost material) has been employed aiming at evaluating the performance of both pure and doped manganese dioxide-based nanomaterials, as electrocatalysts.

## 2. Results and Discussion

### 2.1. Morphological and Structural Characterization of MnO_2_ Nanomaterials

MnO_2_ powders are widely used as cathodic material in batteries [16,17,35]; their electrochemical reactivity generally depends on morphological (surface area, size and type of pores, particle size) and structural (crystalline phases, presence of defects (microtwinning and De Wolff disorder) [36,37]) properties. Among the possible MnO_2_ polymorphs, the γ-form, which consists of an intergrowth structure of β-pyrolusite and β-ramsdellite [35,37], generally exhibits the highest electrochemical reactivity [36,37,38]. The physico-chemical properties of γ-MnO_2_ vary considerably with synthetic procedures and experimental conditions [39,40,41]. Herein, we adopted a hydrothermal route to prepare pure and Ag-doped samples.

**Figure 1:**
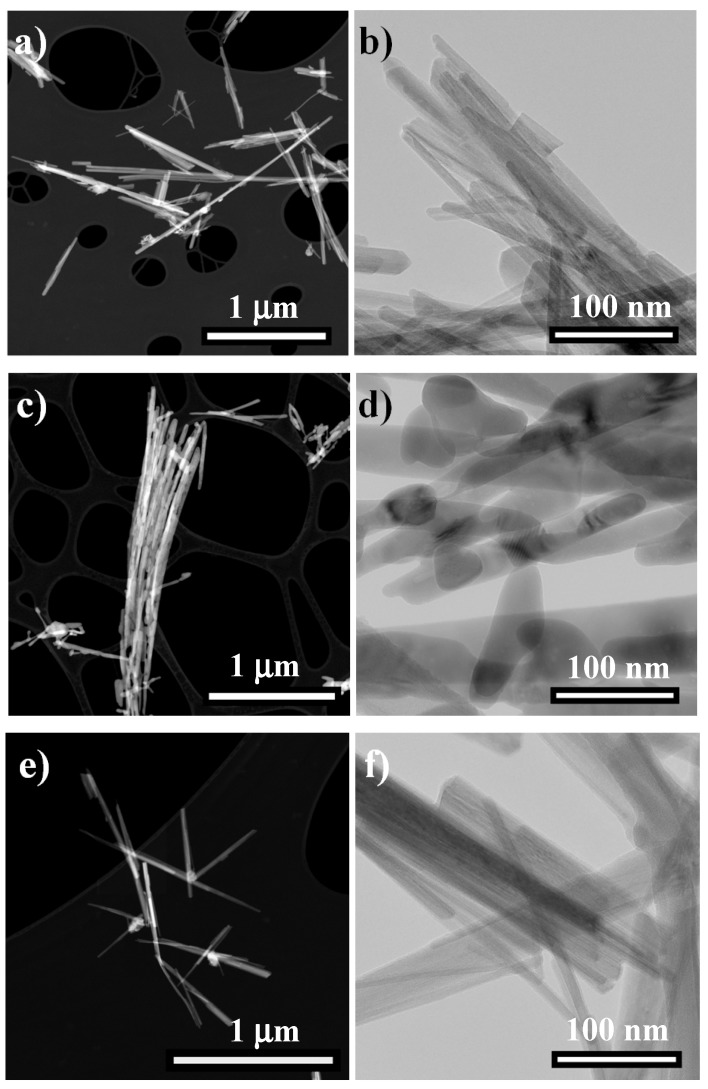
High-angle annular dark field (HAADF)-scanning transmission electron microscopy (STEM) (on the left) and transmission electron microscope (TEM) (on the right) images of (**a**,**b**) hydrothermal MnO_2_ (M_hydro), (**c**,**d**) M_500 (500 °C) and (**e**,**f**) M_hydro_1.0%Ag samples.

The SEM image of hydrothermal MnO_2_ (M_hydro) (Figure S1a) shows the micrometric spherical aggregates (2–5 μm) of MnO_2_ composed by nanosized tiny sticks, with diameters in the range of 20–50 nm and lengths up to several hundred nanometers (Figure 1a,b). This result is fully in agreement with what was reported by Benhaddad *et al.* [38], who synthesized powders consisting of assembled straight needles characterized by similar average sizes. A further thermal treatment leads to the formation of bigger aggregates (up to 10 μm), as can be seen in Figure S1b in the case of particles calcined at 500 °C. After the thermal treatment, the elongated shapes are retained (Figure 1c,d), but their morphology strongly changes: several structural defects appear and the needles, characterized by greater sizes, become smoother with respect to the bare ones.

The surface area, the total pore volume and the relative diameter of mesopores between 6 and 20 nm are reported in Table 1 for all of the synthesized nanomaterials.

**Table 1:** Specific surface area (*S_BET_*), total pore volume (*V_tot. pores_*) and relative percentage of the pore size with a diameter (*d*) between 6 and 20 nm for hydrothermal, calcined and doped MnO_2_ samples.

Sample	*S_BET_* (m^2^·g^−1^)	*V_tot. pores_* (mL·g^−1^)	6 nm < *d* < 20 nm
**M_hydro**	97	0.336	49
**M_200**	70	0.391	40
**M_300**	61	0.360	39
**M_400**	46	0.310	19
**M_500**	28	0.290	2
**M_hydro_0.5%Ag**	88	0.325	51
**M_hydro_1.0%Ag**	75	0.300	50
**M_hydro_2.0%Ag**	73	0.248	49

**Figure 2:**
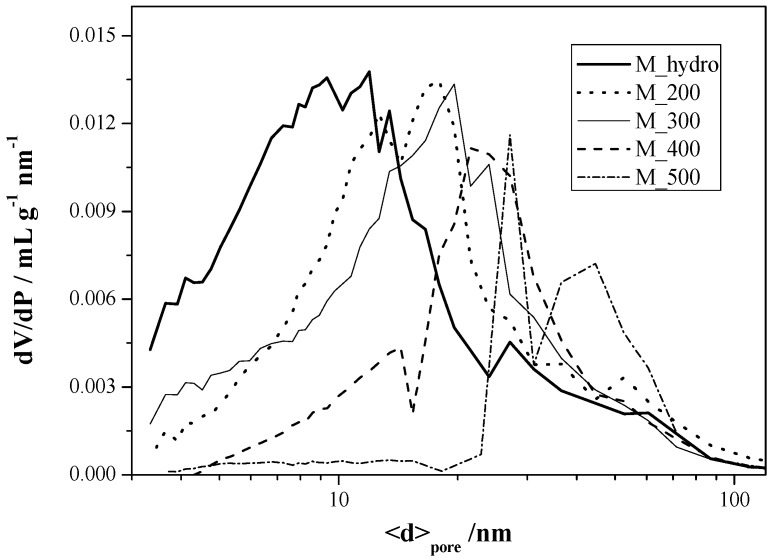
Comparison of the pore size distribution (calculated by the Barrett Joyner Halenda (BJH) method) of MnO_2_ powders at increasing calcination temperature.

The hydrothermal powder (M_hydro) shows the highest surface area (97 m^2^·g^−1^) and the largest amount of pores with *d* < 20 nm, a typical porosity among the nanoneedles making the sticks [38]. The calcination temperature provokes a drastic decrease in the surface area of MnO_2_ powders, which is due to the progressive sintering of particles and the collapse of pores, especially for the smallest ones (see Table 1, fourth column). According to these results, it can be noted that the heating step has a great effect on the pore size distribution of the synthesized MnO_2_ powders. In order to elucidate this effect, Figure 2 shows the pore size distribution for the present samples determined by the Barrett Joyner Halenda (BJH) method. The average pore size shifts from 10 nm for the hydrothermal sample to a higher value (around 20–30 nm) for the samples calcined in the range of 200–400 °C. At 500 °C, a second population appears (~80 nm) corresponding to pores between sticks observed inside the MnO_2_ bowls, as already reported in the literature [38].

**Figure 3:**
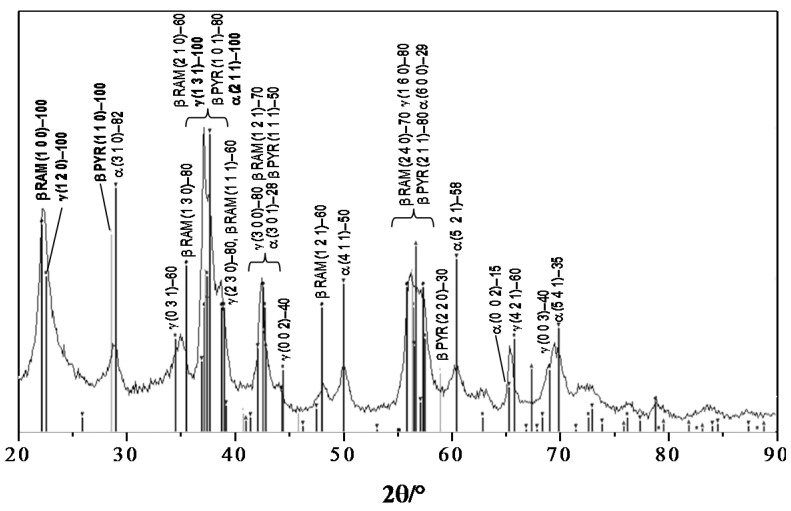
X-ray powder diffraction (XRPD) pattern of the M_hydro sample with the most intense reflections (*hkl*, intensity) of the main polymorphs (γ-MnO_2_ nsutite, β-MnO_2_ ramsdellite (RAM), β-MnO_2_ pyrolusite (PYR) and α-MnO_2_ hollandite).

From the structural point of view, many polymorphs are present in the M_hydro powder. Notwithstanding the absence of a heating procedure, the sample is well crystallized due to Ostwald ripening mechanisms of dissolution/precipitation typical of hydrothermal growth. Figure 3 shows this complex scenario, characterized by the presence of several crystalline phases. All of the reflections (with the *hkl* values and the principal relative intensity) of the X-ray diffraction line can be mainly indexed to four crystallographic polymorphs: (i) γ-MnO_2_ nsutite (International Centre for Diffraction Data Powder Diffraction File (ICDD PDF-2) Card No. 14-0644) [36]; (ii) β-MnO_2_ ramsdellite (ICDD PDF-2 Card No. 04-0378) [42]; (iii) β-MnO_2_ pyrolusite (ICDD PDF-2 Card No. 04-0591) [43]; and (iv) α-MnO_2_ hollandite (ICDD PDF-2 Card No. 44-0141) [44].

**Figure 4:**
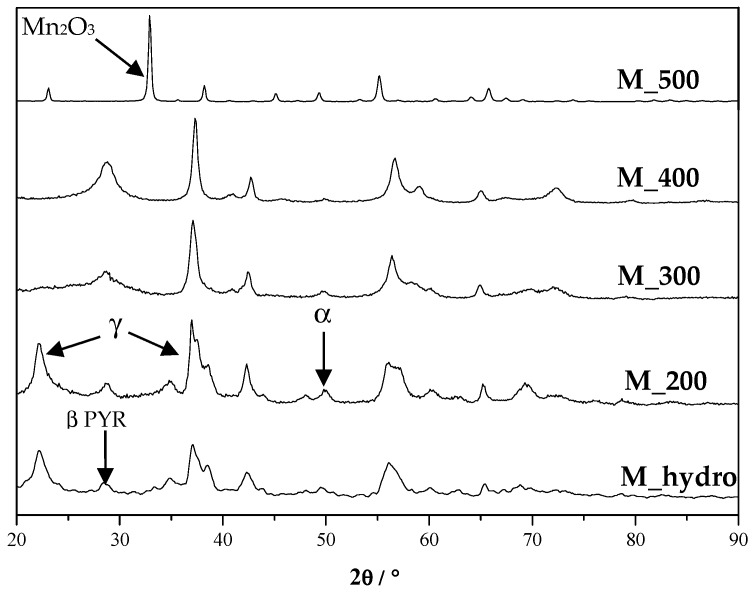
XRPD patterns of MnO_2_ samples calcined at different temperatures. The most significant reflections for γ-MnO_2_ nsutite, β-MnO_2_ pyrolusite, α-MnO_2_ hollandite and Mn_2_O_3_ bixbite are highlighted.

Figure 4 shows that high calcination temperatures (*T* > 300 °C) lead to an increase of β-pyrolusite (100% reflection at around 28°, characterized by a low fraction of defects [38]) content to the detriment of the more electrochemically-active γ-MnO_2_ form. At around 28°, also the α-MnO_2_ polymorph could be present (the 310 plane, see Figure 3), but at higher temperatures, small traces are appreciable (see the peak at 50° ascribable only to the 411 reflection of α-MnO_2_ phase). Finally, at 500 °C, a new phase appears indexed as bixbite Mn_2_O_3_ (ICDD PDF-2 Card No. 71-0636) [45]. On the basis of both structural and morphological results, it can be affirmed that the heating temperature engenders the transformation of γ-MnO_2_ to β-pyrolusite and provokes the decrease of the surface area and pore volume, leading to higher crystallinity of the samples. Thus, for the evaluation of the performances of the final Li/Air battery, only M_hydro was used for the fabrication of the GDE cathodes. All of the calcined samples show very poor electrocatalytic activity: particularly, GDE-H-M_200, formed by hollow core mesoporous shell carbon (HCMSC) and MnO_2_ calcined at 200 °C, gives a specific capacity less than 1000 mAh·g^−1^ at the first discharge run, which rapidly decreases, within four cycles, to a value close to zero, losing about 99% of its specific capacity after the first cycle.

Regarding the hydrothermal samples doped with silver ions, three different molar ratio percentages (0.5%, 1.0%, 2.0%) were adopted. All of the samples show lower surface area and pore volume with respect to the undoped ones, but the same pore size distribution (Table 1, fourth column). Furthermore, the addition of Ag leads to phase composition, needles (Figure 1e,f) and an aggregate size (Figure S1c) comparable to the hydrothermal sample. From structural point of view, the increase of Ag content does not modify both the crystallinity and the lattice parameters, as shown in Figure S2, for bare and 1.0% Ag-doped samples (the latter as a representative sample of differently-doped powders). These effects may be due to a mild modification in the MnO_2_ lattice parameters, induced by Ag atoms. Among the dopant concentrations, EDX mapping (Figure S1d) shows that the 1.0% molar ratio (M_hydro_1.0%Ag) can be considered the best homogeneous dopant distribution in the MnO_2_ matrix; for this reason, only the latter powder was used in the lithium-air cell prototype.

### 2.2. Electrochemical Characterization of GDEs

GDEs, listed in Table 2 and prepared using mesoporous carbons (MCC, mesocellular carbon or HCMSC, hollow core mesoporous shell carbon, as supports) and MnO_2_ powders (as the electrocatalysts), were investigated as described in the experimental part. They were tested at least three times in the same conditions in order to highlight both the reproducibility of the preparation route and the effects of the macroscopic morphology and of the surface texture on the electrochemical performances.

**Table 2:** Investigated gas diffusion electrodes (GDEs) with the relevant chemical compositions and active material loading. H: HCMSC (hollow core mesoporous shell carbon); M: MCC (mesocellular carbon); V: Vulcan XC72R; PVDF: polyvinylidene fluoride; SAB: Shawinigan Black AB50 carbon.

Name	Composition	Loading (mg·cm^−2^)
**GDE-V**	PVDF (15%), SAB (20%), Vulcan XC72R (65%)	1.3
**GDE-V-Pt**	PVDF (15%), SAB (20%), 10% Pt-loaded Vulcan XC72R (65%)	1.3
**GDE-M**	PVDF (15%), SAB (20%), MCC (65%)	1.1
**GDE-M-M_hydro**	PVDF (15%), SAB (20%), MCC (45%), M_hydro (20%)	2.2
**GDE-H**	PVDF (15%), SAB (20%), HCMSC (65%)	1.4
**GDE-H-M_hydro**	PVDF (15%), SAB (20%), HCMSC (45%), M_hydro (20%)	1.3
**GDE-H-M_hydro_1.0%Ag**	PVDF (15%), SAB (20%), HCMSC (45%), M_hydro_1.0%Ag (20%)	1.3

**Figure 5:**
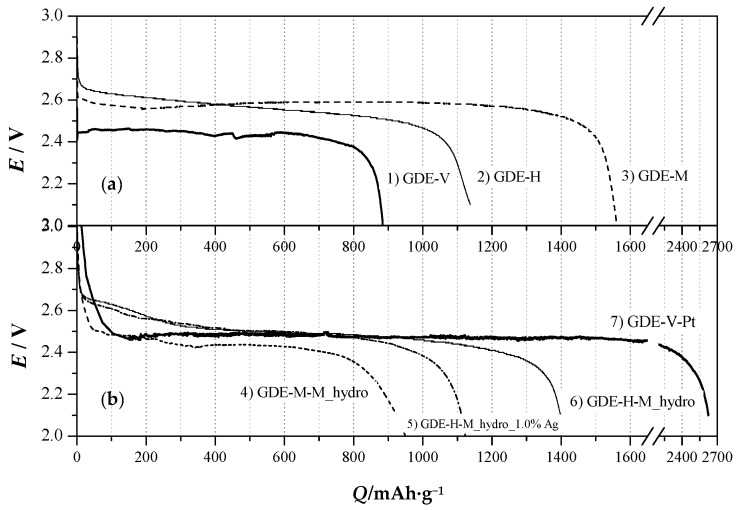
*E vs. Q* curves of GDEs by varying the (**a**) mesoporous carbon matrices and (**b**) carbonaceous supports added with MnO_2_, during the first discharge cycle carried out at 0.34 mA·cm^−2^ in the Swagelok™-type cell (*S-*cell). Bold line: reference GDEs (GDE-V, Curve 1 and GDE-V-Pt, Curve 7).

Figure 5a compares GDEs composed of the different synthesized mesoporous carbon supports and the reference one (GDE-V, Curve 1) during the first discharge cycle, using the *S*-cell configuration (Figure S3a). The reference support, GDE-V (Figure 5a, Curve 1), shows an inhomogeneous discharge profile with a plateau at 2.45 V and a very low specific capacity of about 900 mAh·g^−1^, consistent with the literature data [46]. All of the specific capacities are calculated by dividing the charge amount by active material weight, that is the sum of carbonaceous support and electrocatalytic nanopowders, when present. GDE-H (Figure 5a, Curve 2) and GDE-M (Figure 5a, Curve 3), exploiting HCMSC and MCC carbon matrices, respectively, behave better than either the reference cathodes or the newly-proposed material, like graphene [47,48]. MCC allows reaching a specific capacity of 1560 mAh·g^−1^, while the HCMSC-based cathode gives 1150 mAh·g^−1^. This different behavior is related to the pore distribution characteristics of the material: finer pores, less than 40 Å wide (HCMSC), hinder an easy growth and accommodation of the discharge products (Li-oxides).

Thus, the pores become quickly clogged by Li-oxides, shielding the surface suitable for crystals’ nucleation. On the contrary, MCC, with larger pores (310 Å wide), easily accommodates the discharge products, presenting a larger surface on which nucleation can occur. This has a direct effect on the charge quantity effectively obtainable, thus proving that pore dimensions affect the specific capacity of the battery much more than the specific active area [27]. The performances of Li/air battery cathodes prepared with MnO_2_ electrocatalytic powder added to a mesoporous carbon support are presented in Figure 5b, in which the reference cathode, GDE-V-Pt (Curve 7), is also included. The latter one shows the highest charge density (~2600 mAh·g^−1^) during the first discharge cycle and an efficient discharge potential of about 2.5 V. This extremely high efficiency is due to the ORR (oxygen reduction reaction) electrocatalytic behavior of Pt powder supported on Vulcan, which affects also the plateau discharge potential; the value of 2.5 V *vs.* Li^+^/Li is comparable to that already reported in the literature [49]. However, Pt is known to be a metal that prevents the further reaction of the discharge products (Li_2_O and Li_2_O_2_), thus inhibiting the cyclability of the entire cell. Actually, in the case of GDE-V-Pt, a subsequent deactivation together with a dramatic decrease of the specific capacity after the first cycle occurs. Instead, the addition of the electrocatalytic M_hydro to the mesoporous carbon support reduces significantly the specific capacity furnished by MCC-based cathodes. Indeed, comparing GDE-M (Figure 5a, curve 3) and GDE-M-M_hydro (Figure 5b, curve 4), a loss of about 600 mAh·g^−1^ of the specific capacity can be noticed. This effect is probably ascribed to the pore size of the mesoporous support, which can be partially clogged by the MnO_2_ powders, hence reducing the active surface area and concomitantly favoring the accommodation of the products of discharge cycles. This phenomenon can also explain the lower discharge potential of GDE-M-M_hydro when compared to GDE-V-Pt (2.4 *vs.* 2.5 V). On the contrary, M_hydro added to HCMSC based cathodes slightly increases the specific capacity of the composite electrodes; actually, comparing GDE-H (Figure 5a, Curve 2) and GDE-H-M_hydro (Figure 5b, Curve 6), an increase of more than 200 mAh·g^−1^ can be observed. Probably, the presence of mesoporosity in the MnO_2_ samples, characterized by an order of magnitude higher than HCMSC distribution, does not allow any occlusion processes. Moreover, the larger surface area, the morphology of HCMSC and the homogeneous distribution of MnO_2_ allow a better electron transfer process, thus making this GDE comparable to GDE-V-Pt, in terms of discharge potentials (2.5 V). It is worth noting that the addition of MnO_2_ may catalyze the removal of LiO_2_ and Li_2_O_2_ from the cathode surface during the charge cycles, thus increasing the durability and the cathode performances.

Finally, by analyzing the electrocatalytic performances of GDE-H-M_hydro_1.0%Ag (Figure 5b, Curve 5) and GDE-H (Figure 5a, Curve 2), the former seems not to extremely affect the HCMSC specific capacity, even if the *Q* value is 300 mAh·g^−1^ lower than that in the case of pure MnO_2_ nanopowders.

**Figure 6:**
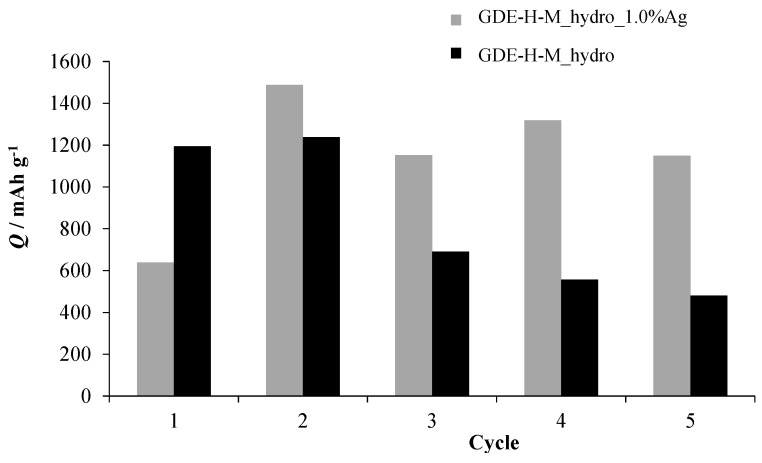
Specific capacity of GDE-H-M_hydro and GDE-H-M_hydro_1.0%Ag during the first five cycles in the home-made cell (*H*-cell) (black and grey histograms, respectively).

For cyclability tests, an *H*-cell with a large volume has been employed in order to avoid electrolyte limitation due to its possible degradation [28,29,30]. In Figure 6, the specific capacity of GDE-H-M_hydro and GDE-H-M_hydro_1.0%Ag during the first five discharge cycles is reported. According to the literature [50,51,52,53], the first cycles contain the key information for a rapid screening of the electrode materials in terms of cell performances. Indeed, the chemical and morphological modifications of various composite electrodes [50,51,52] are comparable, since the limitations connected with the slow kinetics of the oxygen reactions [53] could be overcome. After the fifth cycle, the specific capacities of GDEs, prepared using all of the synthesized nanopowders, remain constant for the other five cycles. Unfortunately, when all of the air cathodes were stressed after ten cycles, the discharge capacity fades.

**Figure 7:**
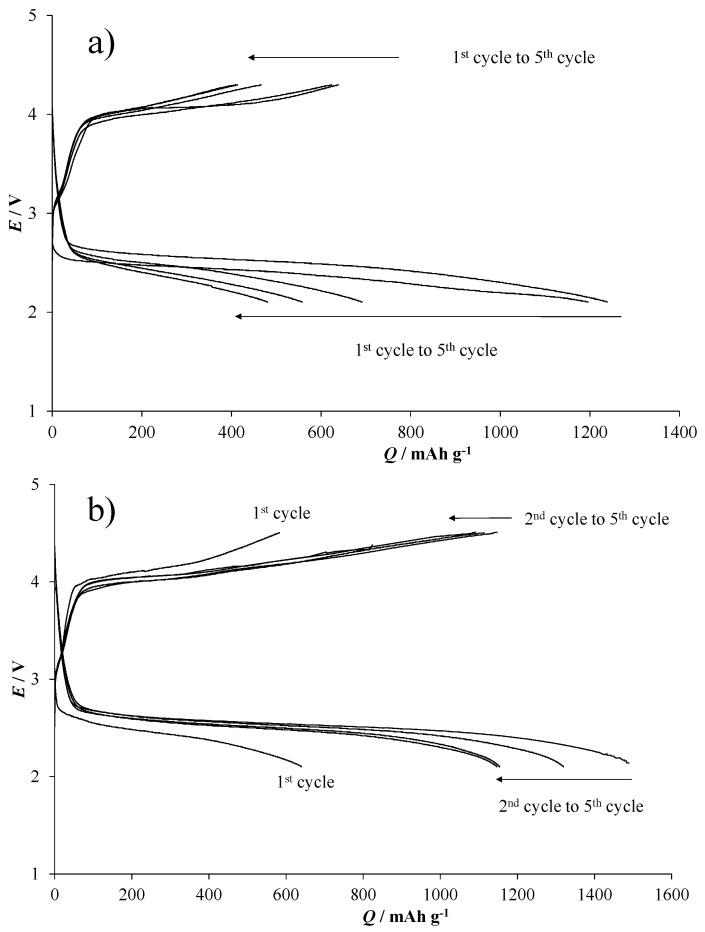
Discharge and charge cycles for (**a**) GDE-H-M_hydro and (**b**) GDE-H-M_hydro_1.0%Ag in the *H*-cell.

GDE-H-M_hydro, prepared using HCMSC carbon support with pure MnO_2_, shows a dramatic decrease (around 50%) in specific capacity after the second run, highlighting the incomplete recharge processes by cycling consecutively. Instead, GDE-H-M_hydro_1.0%Ag shows a specific capacity up to 1400 mAh·g^−1^, remaining above 1100 mAh·g^−1^ for all of the following cycles. Generally, the increase of specific capacity between the first and the second cycle for both cathodes can be ascribable to activation processes that allow a better accommodation and an easier removal of the solid discharge products. In particular, this effect is more evident for GDE-H-M_hydro_1.0%Ag (Figure 6, grey histograms) due to the presence of Ag dopant in the electrocatalytic powder, which plays an active role for the battery charge/discharge processes. Indeed, the addition of silver species leads to larger electrochemically active sites of the working material [54,55] and to transporting the insulating Li oxide products away from the carbon surface, in order to prevent it from blocking the electron transfer needed to reduce the O_2_ [22]. The same behavior is underlined by the discharge/charge curves (*E vs. Q*, Figure 7) carried out at 0.34 mA·cm^−2^.

While GDE-H-M_hydro (Figure 7a) shows a rapid decrease of the specific capacity by cycling, in GDE-H-M_hydro_1.0%Ag (Figure 7b), a possible synergistic effect of MnO_2_ and Ag occurs. In the present case, a stabilization of the discharge and charge curves above 1100 mAh·g^−1^ (from the third cycle on) for GDE-H-M_hydro_1.0%Ag is evident, as already seen in Figure 6. Moreover, the cut-off charge potential value for GDE-H-M_hydro is 4.3 V; at this potential, all of the tested cathodes undergo a rapid decrease of the performances, probably due to the presence of undesired discharge products. The other way around, the cut-off charge potential up to 4.5 V without any evidence of cathode degradation is noteworthy for GDE-H-M_hydro_1.0%Ag, pointing to the capability of Ag-doped MnO_2_ electrocatalyst to easily remove the solid discharge products from the GDE pore structure.

**Figure 8:**
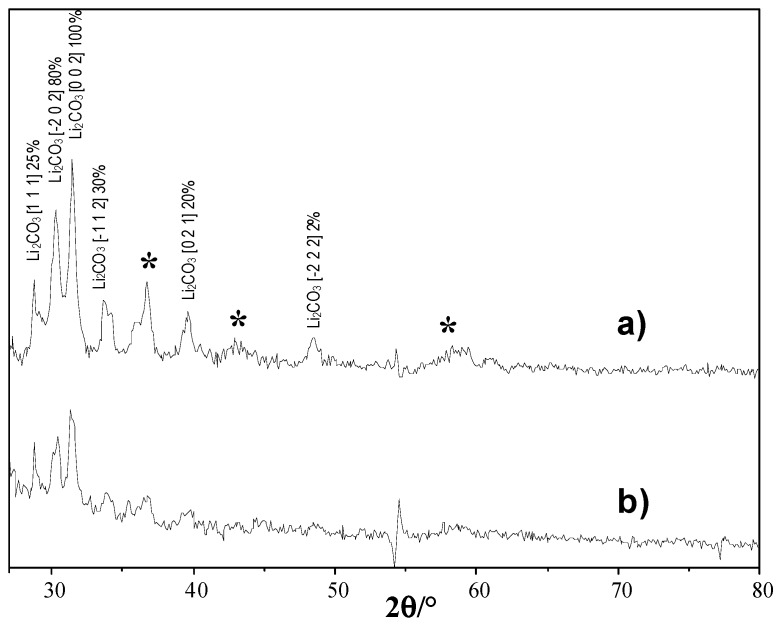
X-ray diffraction lines of (**a**) GDE-H-M_hydro and (**b**) GDE-H-M_hydro_1.0%Ag obtained by subtraction between the GDEs after five cycles and before cycling. The * label indicates the presence of MnO_2_ polymorphs.

These outcomes can be also appreciated looking at Figure 8a,b, in which the X-ray diffraction lines of GDE-H-M_hydro and GDE-H-M_hydro_1.0%Ag (obtained by subtraction of the respective patterns before and after five cycles) are reported. In all cases, the signals due to Li_2_O_2_ are almost absent for both GDEs; however, the presence of Li_2_O (ICDD PDF-2 Card No. 077-2144) for both GDEs and of Li_2_CO_3_ (ICDD PDF-2 Card No. 022-1141), particularly for GDE-H-M_hydro (Figure 8a), is appreciable. Bruce *et al.* [26] discussed both the absence of Li_2_O_2_ and the presence of insoluble Li_2_CO_3_ coming from the degradation of carbonate solvents. In our case, the low amount of Li_2_CO_3_ for GDE-H-M_hydro_1.0%Ag justifies the less oxidative degradation of carbonate solvent and an easier removal of the discharge products from the GDE surface, probably due to the increased electronic conductivity of Ag-doped MnO_2_ [22]. Actually, the presence of electrocatalytic material in GDE could help to reduce the solvent degradation [34], thus evidencing the possibility of a further increase of the solvent stability. Nowadays, the electrochemical stability of these compounds must be better investigated for their use in Li/Air devices, especially in the presence of suitable additives (vinylene carbonate, ethylene sulfite, *etc.*) able to stabilize the solvent [33], limiting degradation phenomena.

## 3. Experimental Section

### 3.1. Cathode Material and Gas Diffusion Electrode

In the present paper, specific attention has been given to cathodic materials. A general criterion in preparing GDEs was kept throughout all of the experiments: 20% by weight of manganese dioxide was added to a mixture of carbonaceous matrix (see the following). MnO_2_ samples (see Table 1) were prepared via hydrothermal synthesis, operating a redox reaction of manganese sulfate with potassium permanganate (1:1 molar ratio) in 15 mL of Milli-Q (Darmstadt, Germany) water; reactants were mixed together and kept under stirring conditions at 90 °C for 24 h [38,56]. As a result, black powders were obtained, which were filtered, rinsed with Milli-Q water several times and finally dried at 60 °C for 24 h to provide the complete drying of MnO_2_ (labeled as M_hydro in the following). A quote of pure MnO_2_ was further subjected to a thermal treatment after the hydrothermal synthesis in the range 200–500 °C for 5 h in N_2_ flux (named M_200 → M_500). A third group of samples was prepared to overcome the resistive behavior of manganese dioxide by adding AgNO_3_ to the reaction mixture during the hydrothermal growth by varying the Ag/Mn molar ratio from 0.5% to 2% (called M_hydro_*x*Ag, where *x* = molar ratio %). Zhang *et al.* [57] demonstrated that the increase in the conductivity leading to higher specific capacitance is due to the addition of silver into MnO_2_ matrix.

Carbonaceous supports, a mesocellular carbon (MCC) and a hollow core mesoporous carbon (HCMSC), were prepared as widely described in the literature [46,58,59,60]. MCC shows a BET area of 231 m^2^·g^−1^ and a pore size of 310 Å, while HCMSC has a higher BET area, 1150 m^2^·g^−1^, with pores one order of magnitude smaller (38 Å). Empty HCMSC core shell particles create an extremely regular pattern of 400–500 nm diameter spherules.

GDEs (see Table 2) were prepared by mixing in N-methyl-2-pyrrolidinone (NMP 99.5%, anhydrous used as solvent, Sigma-Aldrich^®^, Milano, Italy), the mesoporous carbon and the manganese dioxide together with a small percentage by weight of Shawinigan Black AB50 carbon (SAB, Chevron Phillips, The Woodlands, TX, USA) and polyvinylidene fluoride (PVDF Kynar^®^, Arkema, Barcelona, Spain) powder, employed as a binder. The relative weight ratio among the components was: (MCC or HCMSC):SAB:PVDF = 65:20:15 or (MCC or HCMSC):MnO_2_:SAB:PVDF = 45:20:20:15. After a 3-h stirring period, the slurry was brush-deposited, at 55 °C, onto a carbon paper sheet (SIGRACET^®^, SGL Group, Meitingen, Germany). The complete evaporation of NMP was obtained by leaving the GDEs in an oven at 60 °C for 12 h. The electrode was punched into a 1.17-cm^2^ disk with 1.3–2.2 mg·cm^−2^ of active material (excluding PVDF, the binder). Vulcan XC72R and Vulcan XC72R with 10% Pt were used to prepare GDEs to be tested as reference cathodes for the involved reactions. The catalytic activity and the physical characteristics of the carbon materials strongly affect the performance and the life of the electrodes. Maja *et al.* [61] reported that the proper selection of carbons leads to a significant enhancement of the performance of gas diffusion electrodes. Particularly, at long operation times, cathodes with Shawinigan acetylene black show better performances than those with oil-furnace carbon supports. Furthermore, the combination of Vulcan XC72R and SAB gives the best catalytic performances.

### 3.2. Cell Configuration

A solution consisting of 1 M LiClO_4_ (Sigma-Aldrich^®^, battery grade, dry, ≥99.99%), dissolved in propylene carbonate (PC: Sigma-Aldrich^®^, anhydrous, 99.7%), was used as the electrolyte. In an argon-filled (Gastec Vesta 5.5) glove box (LABCONCO^®^, Kansas, MO, USA), Li-air Swagelok™-type cells [62] were assembled stacking a Li disk (Sigma- Aldrich^®^, 99.9%) as the counter electrode, a glass fiber separator wetted with 90–100 μL of electrolyte and the gas diffusion working electrode. Concerning the choice of lithium salt, although it has no industrial application due to severe safety issues, LiClO_4_ has been used in several academic studies reporting the high rechargeability of Li-O_2_ cells. The relatively higher stability of LiClO_4_ suggests it continues to be suitable for the purpose of tests and development at the research level [63]. Two different experimental cells were investigated: the first one, a Swagelok™ cell (S-cell) that ensured a rapid assembly, a handy configuration and an easy maintenance; the other one, a home-made cell (*H*-cell), in which the prepared GDEs, used as the cathode, can directly face a massive electrolyte solution (40 mL), in order to avoid the possible electrolyte limitations [27]. The anode was also employed as a reference electrode, and potential values have been always referred to the Li^+^/Li redox couple. Oxygen (Gastec Vesta 5.5) was fed to the gas side of the GDEs at a 1-mL·s^−1^ flow, during both the charging and discharging period. Both of the cells were studied performing chronopotentiometric experiments (galvanostatic analysis) imposing a discharge and charge current density and cut-off potential of 0.34 mA·cm^−2^ and of 4.3 V (*vs.* Li/Li^+^), respectively.

### 3.3. Instrumentation

A Princeton Applied Research 263A potentiostat/galvanostat was employed to perform cycle tests, and CorrWare 3.1c software (Oak Bridge, TN, USA) was used for data acquisition. Room-temperature X-ray powder diffraction (XRPD) patterns were collected between 20 and 90° (Δ2θ = 0.08°, time per step = 17 s, scan speed = 0.02°/s) with a Siemens D500 diffractometer (Berlin, Germany), using Cu K_α_ radiation (Cu K_α1_: λ = 1.54056 Å, K_α2_: λ = 1.54433 Å). Specific surface areas were determined by the classical BET procedure using a Coulter SA 3100 apparatus (BeckmanCoulter, Fullerton, CA, USA). Desorption isotherms were used to determine the pore size distribution using the Barret Joyner Halenda (BJH) method. Particle morphology was obtained by scanning electron microscopy (SEM), using a Hitachi TM-1000 (Tokyo, Japan) equipped with an energy dispersive X-ray (EDS, SwiftED-TM) detector. Transmission electron microscope and scanning transmission electron microscope (TEM/STEM) analyses were performed by a ZEISS LIBRA200 EFTEM instrument (Jena, Germany) operated at 200 kV. The STEM micrographs were collected by a high-angle annular dark field (HAADF) detector. The TEM specimens were prepared suspending the nanoparticles in isopropanol, sonicating the suspension in an ultrasonic bath for 20 min and dropping it onto a holey carbon-coated Cu grid. The specimens were analyzed after complete solvent evaporation in air at room temperature.

## 4. Conclusions

Lithium-air batteries represent valid devices in facing the increasingly worldwide energy demand. Nevertheless, before they can be fully developed and employed in different fields, from automotive to portable devices, cathode optimization is requested together with the development of a new family of low cost electrolytes, able to resist the presence of oxygen radicals. Nowadays, the optimization of cathodic materials is required to develop batteries able to produce high specific energy.

In this work GDE composites, made of two diverse mesoporous carbons (MCC and HCMSC) and bare and Ag-doped MnO_2_ electrocatalysts, have been deeply characterized and electrochemically tested in home-made Li/air cells. All hydrothermally-synthesized MnO_2_ nanoparticles show a nanoneedle structure; particularly, M_hydro presents the best properties in terms of surface area and total pore volume distribution. As concerns the doped samples, only M_hydro_1.0%Ag has been tested due to its homogeneous distribution in the MnO_2_ matrix.

The behavior of the GDE obtained by using only mesoporous carbons depends on their pore size distribution: MCC with larger pores (310 Å) shows a specific capacity of 1560 mAh·g^−1^, while HCMSC (*d* = 40 Å) reaches a value around 1150 mAh·g^−1^. When electrocatalytic M_hydro nanopowders are incorporated into the composite GDE, a drastic decrease of performance can be evidenced for MCC-based cathodes, whereas no effects are significant in the case of HCMSC-based ones. Hydrothermal MnO_2_ doped with Ag 1.0% shows an average specific capacity above 1100 mAh·g^−1^ during cycles. This effect can be due to silver atoms in a manganese oxide lattice that induce an electrical conductivity improvement and limit the presence of Li-based discharge products. The combination of Ag with MnO_2_ could lead to composite cathodes for Li/air batteries that present high specific capacity, thus reducing the overpotentials and increasing the cyclability of the cell.

## Supplementary Materials

They are available online at http://www.mdpi.com/2079-4991/6/1/10/s1.
