# Supplementary material for: The Influence of Carbonaceous Matrices and Electrocatalytic MnO2 Nanopowders on Lithium-Air Battery Performances
Source: Nanomaterials (Basel). 2016 Jan 6;6(1):10. doi: 10.3390/nano6010010 (PMC5302542; doi:10.3390/nano6010010)
Supplement: Supplementary file 1 [file nanomaterials-06-00010-s001.pdf]

Supplementary Materials

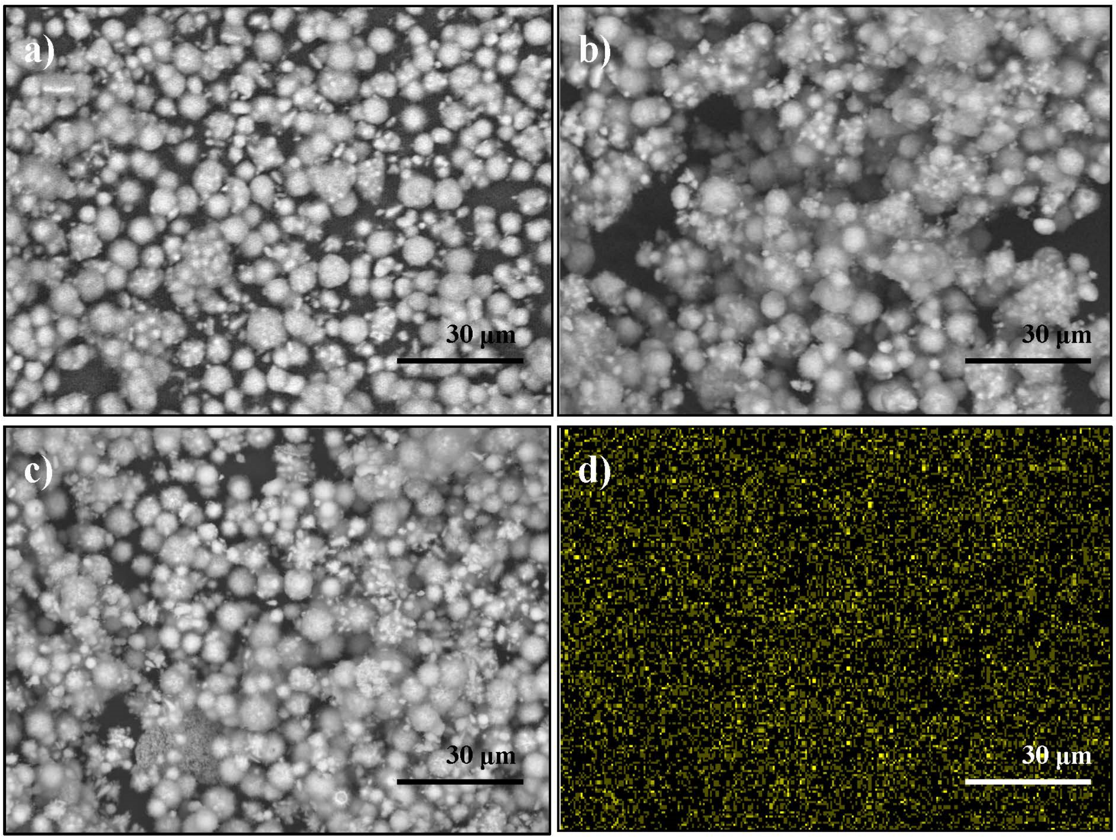

Figure S1. Scanning electron microscopy (SEM) images of (**a**) hydrothermal (M_hydro), (**b**) calcined (M_500) and (**c**) silver-doped (M_hydro_1.0%Ag) samples, (**d**) Energy dispersive X-ray (EDX) mapping of M_hydro_1.0%Ag powder.

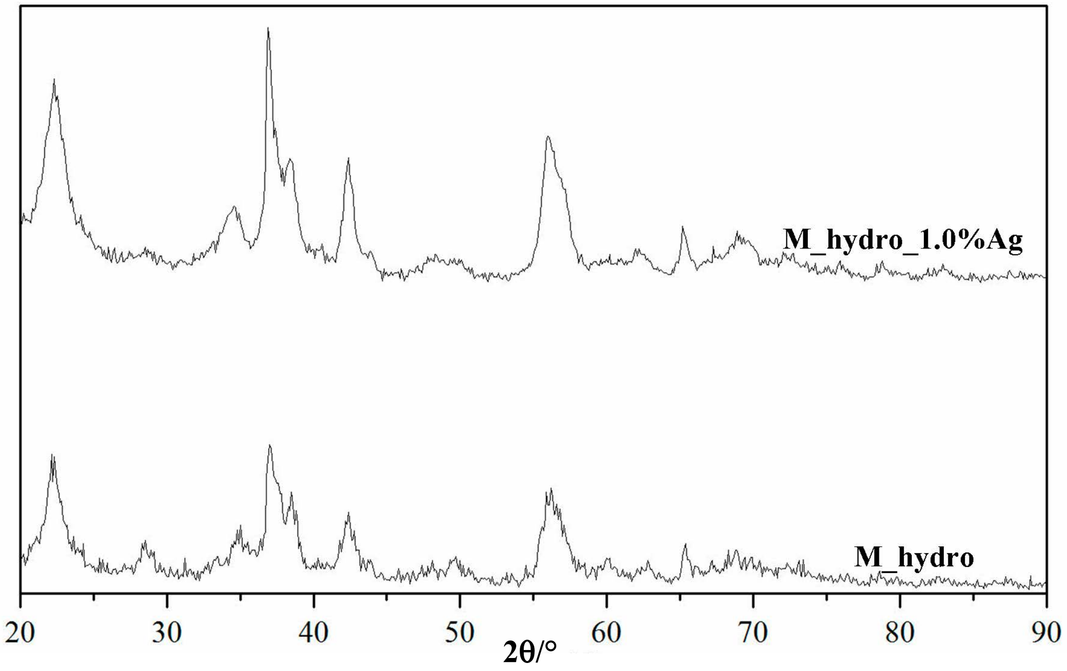

Figure S2. Comparison between X-ray powder diffraction (XRPD) patterns of pure (M_hydro) and Ag-doped MnO_2 (M_hydro_1.0%Ag) samples.

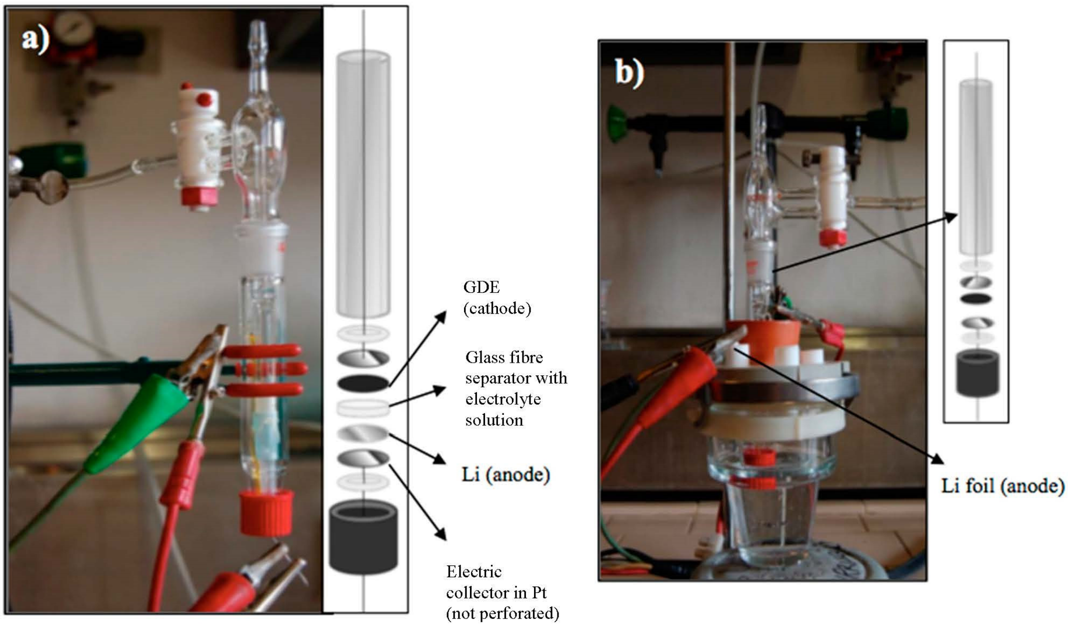

Figure S3. Cell configurations used for the electrochemical tests: (**a**) a Swagelok™-type cell (*S*-cell) and (**b**) a home-made cell (*H*-cell).

© 2016 by the authors; licensee MDPI, Basel, Switzerland. This article is an open access article distributed under the terms and conditions of the Creative Commons by Attribution (CC-BY) license (http://creativecommons.org/licenses/by/4.0/).
